# Patterns of Change in Metabolic Capabilities of Sediment Microbial Communities in River and Lake Ecosystems

**DOI:** 10.1155/2018/6234931

**Published:** 2018-05-27

**Authors:** Adam Oest, Ali Alsaffar, Mitchell Fenner, Dominic Azzopardi, Sonia M. Tiquia-Arashiro

**Affiliations:** Department of Natural Sciences, University of Michigan-Dearborn, 4901 Evergreen Road, Dearborn, MI 48128, USA

## Abstract

Information on the biodegradation potential of lake and river microbial communities is essential for watershed management. The water draining into the lake ecosystems often carries a significant amount of suspended sediments, which are transported by rivers and streams from the local drainage basin. The organic carbon processing in the sediments is executed by heterotrophic microbial communities, whose activities may vary spatially and temporally. Thus, to capture and apprehend some of these variabilities in the sediments, we sampled six sites: three from the Saint Clair River (SC1, SC2, and SC3) and three from Lake Saint Clair in the spring, summer, fall, and winter of 2016. Here, we investigated the shifts in metabolic profiles of sediment microbial communities, along Saint Clair River and Lake Saint Clair using Biolog EcoPlates, which test for the oxidation of 31 carbon sources. The number of utilized substrates was generally higher in the river sediments (upstream) than in the lake sediments (downstream), suggesting a shift in metabolic activities among microbial assemblages. Seasonal and site-specific differences were also found in the numbers of utilized substrates, which were similar in the summer and fall, and spring and winter. The sediment microbial communities in the summer and fall showed more versatile substrate utilization patterns than spring and winter communities. The functional fingerprint analyses clearly distinguish the sediment microbial communities from the lake sites (downstream more polluted sites), which showed a potential capacity to use more complex carbon substrates such as polymers. This study establishes a close linkage between physical and chemical properties (temperature and organic matter content) of lake and river sediments and associated microbial functional activities.

## 1. Introduction

Lake Saint Clair and the Saint Clair River are important resources for about six million U.S. and Canadian residents who live close to the watershed [1, 2]. The lake and river are defining natural features of Southeast Michigan and Southwestern Ontario and are used for fishing, recreational boating, drinking, and commercial navigation. They are also dynamic parts of the larger Great Lakes system. Lake Saint Clair and the Saint Clair River connect between the upper and lower Great Lakes and are used for both commercial navigation and fish and wildlife that live in or move across the area [3]. They are the outlet for the three upper Great Lakes, with more than 90% of the average annual water supply to Lake Erie and nearly 75% of the supply to Lake Ontario passing through the corridor. Much of the shoreline on both sides of the Saint Clair River is urbanized and heavily industrialized; hence, the pollutants from day-to-day human activities and the industry have contributed to the impairment of the water quality in the lake and river [4]. Due to intensive industrial development that has occurred in and near adjacent cities at the northern end of the river, Lake Saint Clair has become the sink for particles and particle-associated chemical pollutants that enter via the Saint Clair River and the tributary streams.

Pollutants in the Saint Clair River and Lake Saint Clair sediments include toxic organic compounds such as organochlorine insecticides, PCBs, chlorobenzenes, chlorotoluenes, and chlorostyrenes [2, 5–7]. Heavy metals such as copper, nickel, zinc, lead, cadmium, chromium, and mercury have also been detected in the sediments [2, 7, 8]. Accordingly, it is essential to protect and improve the lake and river systems. These necessitate understanding the role of contaminants with more focus on how microbial communities and ecological functions respond to pollution stress [9, 10]. Compared to the surface interstitial waters, sediments are more stable and less variable and serve as a medium for active biogeochemical processes [11–15]. Studies have shown that sediments are considered a reliable recorder of ecological responses to pollution [10, 16–20]. Sediment microorganisms play key roles in nutrient cycling, heavy metal immobilization [21–23], and degradation of organic compounds [24]. As such, they can be used as a potential bioremediation strategy to overcome pollution problems associated with a local river or lake [8, 22, 25, 26]. However, their composition and activity are known to be sensitive to environmental pollutants [24, 27]. For instance, nutrients and heavy metal contamination have been shown to cause changes in bacterial biomass, diversity, and function [21]. These changes can lead to the replacement of more sensitive species by more tolerant species or pollutant-degrading microorganisms [22].

Although microbial communities are an important metabolic component of river and lake sediments, their metabolic versatilities are poorly understood, owing to complicated environmental stresses and regional microbial differentiation. Therefore, physiological profiling of microbial populations is an important subject for a better understanding of the biodegradation potential of the sediment communities and improvement of watershed management. One commonly used approach in environmental microbiology is the community-level physiological profiling (CLPP) using Biolog EcoPlates, which is based on assessing the ability of microbial communities to metabolize a broad range of organic substrates [28]. Although this method has several well-documented limitations, normalization of the data and standardization of the protocol provide robust results [29]. The Biolog EcoPlates have been successfully documented to plankton [17, 30], sediment [31, 32], sediment-water interface [33], groundwater [17], soil [34], and compost [35] microbial communities. In the present study, we optimized the Biolog EcoPlate data processing to overcome some of the shortcomings of this method associated with the use of sediment samples.

Here, we investigated the carbon substrate utilization patterns of the sediment microbial communities, along the Saint Clair River and Lake Saint Clair. The main goal of this study was to determine the potential of CLPP assay for resolving the metabolic diversity of river and lake sediments on temporal and spatial scales and to establish a close linkage between physicochemical properties of the sediments and the associated microbial functional activities. Six sampling sites (three lake sites and three river sites) were chosen to reflect spatial differences. These sites were sampled in the spring, summer, fall, and winter of 2016. The metabolic fingerprints obtained from the samples were used to understand the functional diversity inferred by the carbon substrate shifts among sites of contrasting urbanization.

## 2. Materials and Methods

### 2.1. Site Description

**Figure 1:**
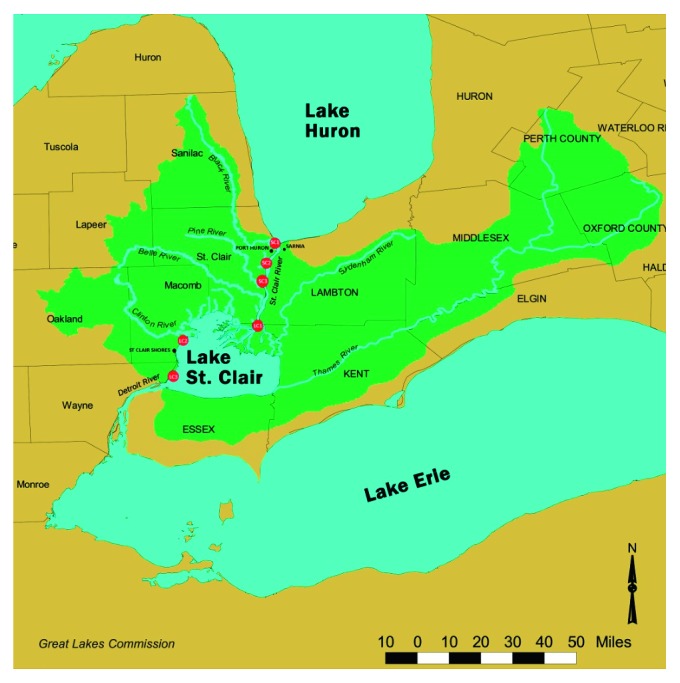
Sediment sample location map for the 2016 sampling. This map shows the Saint Clair River, Lake Saint Clair, and surrounding rivers and cities. The red circles represent the sites where the samples were taken from.

Lake Saint Clair is a shallow productive lake in the drainage system between Lake Huron and Lake Erie (Figure 1). It is the smallest lake in the Laurentian Great Lakes system with a maximum length of 43 km, a mean depth of 3 m, and a surface area of 1115 km^2^ [3]. Although not designated as the Area of Concern by the International Joint Commission, it is situated between two major Areas of Concern, the Saint Clair River and the Detroit River (Figure 1). Because the lake is vulnerable to the potential impact from the Saint Clair River, it was included in the Upper Great Lakes Connecting Channels Study. About 98% of the water flowing into the lake is from the Saint Clair River in the north originating from Lake Huron, while the south is connected to the Detroit River, which drains into Lake Erie (Figure 1). The Thames River, Sydenham River (Ontario, Canada), and Clinton River (Michigan, USA) are the main tributaries of the lake and are enriched with nutrients from land drainage and domestic sewage.

The Saint Clair River is the direct outlet of Lake Huron, and it flows approximately 64 km in a southerly direction to Lake Saint Clair (Figure 1) and forms part of the international boundary between Canada and the United States [4]. Sediments transported along the Saint Clair River substrate consist of eroding glacial clays and medium-to-coarse sands and gravels [2, 36–38]. The river drops almost 1.5 m from the elevation of Lake Huron to that of Lake Saint Clair. It is a relatively straight channel with artificial structures, such as riprap and retaining walls, some narrow beaches, and vegetated cliffs [38].

### 2.2. Sampling Locations and Sampling Procedure

Sediment samples were retrieved at three locations from Lake Saint Clair (LC1, LC2, and LC3) and three locations from the Saint Clair River (SC1, SC2, and SC3) (Figure 1). These locations are in the vicinity of known point sources (e.g., City of Saint Clair permitted wastewater discharges, marina, major river follow, and public parks) to adequately represent source areas' upstream (SC1, SC2, and SC3) and downstream (LC1, LC2, and LC3). The site SC1 (N 42° 58.393; W 82° 25.141) is located upstream where the water from Lake Huron drains into the Saint Clair River; the site SC2 (N 42° 54.323; W 82° 28.036) is close to the City of Marysville Wastewater Treatment Plant, City Public Park, and public boat launch; and the site SC3 (N 42° 49.2′9″; W 82° 29.2′0″) is situated at the mouth of the Pine River, which empties into Lake Saint Clair. The site LC1 (N 42° 37.8′8″; W 82° 30.9′0″) is located downstream of the Saint Clair River, where it empties its water into Lake Saint Clair and is close to the residential private boating docks; the site LC2 (N 42° 31.6′0″; W 82° 52.2′8″) is close to the Veterans Memorial Park located to the south of the Clinton River; and the site LC3 (N 42° 21.560; W 82° 55.5′7″) is situated where Lake Saint Clair empties its water into the Detroit River at the City of Grosse Pointe Park.

A Ponar grab sampler was used to collect sediment samples. The sampling equipment was decontaminated after each sample was processed. The decontamination procedure included rinsing the sampling pans and spoons with site water and using new sampling tubes and gloves for each sample location. A total of 24 sediment samples were collected from the shore of Lake Saint Clair and the Saint Clair River (Figure 1) in the spring (April 18), summer (June 20), fall (September 16), and winter (December 12) of 2016. About 1 kg of sediments was taken from each site throughout 2016. All samples were collected and stored in Ziploc bags, cooled to 4°C in a cooler containing ice, and stored in darkness. The samples were kept in a cooler and transported to the lab. Upon arrival, the samples were stored in a refrigerator for 24 h before any assay began.

### 2.3. Field Analysis

Weather history (air temperature, moisture precipitation, sea-level pressure, and wind) as well as the reference water depth and water elevation was obtained from the NOAA's National Ocean Service website (http://www.coops.nos.noaa.gov/data_res.html). Water pH and water temperature at each site were measured using Horiba's U-10 Water Quality Checker in the field. Water depth and water clarity were measured by using a Secchi disk.

### 2.4. Physicochemical Analysis

Sediment samples were analyzed for moisture content (at 105°C for 24 h) on a dry weight approach (wet weight of the water divided by the dry weight of the sediments). Thus, if the wet weight of the water is greater than the dry weight of the sediments, then the moisture content would be greater than 100%. This is not surprising, as organic materials (some soils) can hold four times their weight in water. Sediment samples were also assayed for ash and organic matter contents using the loss-on-ignition technique (at 550°C for 5 h), pH (1 : 10 water extract; Oakton 500 Series pH meter, Oakton Instruments, Vernon Hills, IL), and electrical conductivity (1 : 10 water extract; Hanna H19810 pH meter, Hanna Instruments, Woonsocket, RI). Particle size distribution of the sediments was determined using the Bouyoucos hydrometer method [39].

### 2.5. Total Bacterial and Fungal Counts and Dehydrogenase Activities

Quantitative estimations of the populations of total aerobic heterotrophs and fungi in the sediment samples were determined by direct plating on appropriate media [40, 41]. The sediments underwent serial dilutions and were spread directly on plate count agar (for heterotrophic bacteria) and rose bengal agar supplemented with 30 *µ*l·ml^−1^ streptomycin (for fungi). Heterotrophic bacterial and fungal plates were incubated at room temperature, but the fungal plates were incubated longer (7 days) than the bacterial plates (48 h). After incubation, visible colonies were counted for each plate and the colony-forming units (CFU g^−1^ dry weight) of the three replicates were computed.

The dehydrogenase activity in the sediments was analyzed using a colorimetric method [42]. Briefly, sediment samples each containing approximately 6 g of oven-dried soil equivalents were taken from 500 ml separate replicates. To each jar, 67 mg of CaCO_3_, 1 ml of a 3% (w/v) aqueous solution of 2,3,5-triphenyl tetrazolium chloride (TTC), and 2.5 ml of distilled water were added. The contents were then mixed to exclude as much trapped air as possible. The mixtures were then incubated in the tubes for 7 days at room temperature. On completion of the incubation, the triphenyl formazan (TPF) formed by the reduction of TTC was extracted with 10 ml methanol. The extract was filtered, and the filtrate was diluted with methanol to a final volume of 50 ml in a 50 ml volumetric flask. The absorbance at 485 nm was measured using a spectrophotometer with methanol as a blank. Soil dehydrogenase activity, expressed as mg of TPF produced g^−l^ of sediments, was estimated by comparison of absorbance values to a standard curve of 0–1000 mg of TPF·ml^−1^ in methanol. Separate absorbance measurements of the methanolic sediment extracts were made without added TTC or TPF, which served as blanks. Values of dehydrogenase are expressed as mg of triphenyl formazan (TPF) released g^−1^ of dry sediments and are presented as an average of three replicates.

### 2.6. Biolog EcoPlate Assay

Biolog EcoPlate™ (Biolog, Inc., Hayward, CA) was used to determine the “metabolic fingerprint” of heterotrophic microorganisms present in the sediment samples based on the carbon source they utilized. Biolog EcoPlate is composed of 31 different carbon compounds and a control. It contains three replicates of the carbon source and control wells. A redox dye (tetrazolium violet) was added in each well, which turns purple when the carbon source is used by the microbial communities present in the sample. As a particular carbon source is used, the color of the well changes. To determine metabolic diversity, the absorbance of the wells is measured in a spectrophotometer or a plate reader [29]. For this assay, 1 : 10 sediment extracts were prepared by placing 5 g of each sediment sample into a 50 ml plastic centrifuge tube (Falcon) containing 45 ml of 0.9% saline solution. The tubes were then placed in a shaking incubator and shaken at 250 rpm for one hour at room temperature in order to extract the heterotrophic microbial communities from the sediments. After shaking, Whatman filter #1 was used to filter out any particulates that may hinder the assay.

Using an automatic micropipette, 125 *µ*l of the filtered extract was dispensed into every well on the corresponding EcoPlate, except for the control (blank) wells, which received 125 *µ*l of 0.9% saline. Color development in the microplate wells was measured at 595 nm using a Tecan Sunrise microplate reader (Tecan, Research Triangle Park, NC). Biolog EcoPlates from the spring, summer, fall, and winter sampling were read at 0, 24, 48, and 72 h in order to generate a relationship between average well color development and time. The plates were incubated at 12°C (spring samples), 25°C (summer samples), 20°C (fall samples), and 8°C (winter samples) in between measurements. Because the samples were cloudy even after filtration, a background correction had to be done, meaning that the measurements done at the time of 24, 48, and 72 hours had to be subtracted from the initial measurements, giving a corrected absorbance. From this corrected absorbance, the average well color development (AWCD) was taken. The AWCD is the average of the readings at 595 nm of all the wells at a particular time. The closer the value to 0.75, the better the AWCD, as 0.75 is regarded to be the best solution because at this value, the response of a microbial community can be seen in most wells and the wells with the most active microbial communities reach the asymptote of color development [29]. Samples with AWCD readings closest to 0.75 were used in statistical analysis. The relative abundance of the different substrates was determined based on the amount (the AWCD value) of substrates utilized relative to the total amount of substrates (the sum of AWCD values of each plate) used by the heterotrophic microbial communities, tested based on the measured densities of reduced tetrazolium dye in wells. The pattern of utilization was calculated using the average of the absorbencies of a particular carbon source and dividing it over the sum of all the absorbencies.

### 2.7. Statistical Analyses

All experiments were carried out in triplicates. Data from three replicate Biolog EcoPlates were used for the statistical assay. Several statistical analyses were performed with the data. The first was an unpaired two-sample *t*-test that compared the river and lake sediments. Analysis of variance (ANOVA) tests were used to compare the three river sites (SC1, SC2, and SC3) and the three lake sites (LC1, LC2, and LC3). A separate ANOVA test was also carried out to compare the carbon utilization patterns of the microbial communities at different seasons (spring, summer, fall, and winter) for the river and lake sediments. When ANOVA showed a significant difference between sites or between seasons (*P* ≤ 0.05), means were separated using Tukey's honest significant test.

Principal component analysis (PCA) was used to determine differences between patterns of the substrate utilization profiles. This statistical analysis reduced the number of variables to a smaller number of variables called principal components (PCs). To compare the utilization profiles, the samples were compared with the 31 variables (C substrates). This was accomplished by projecting the original data onto new axes, or PCs. PC1 accounts for most of the variance of the original sample, and PC2 accounts for the next greatest amount of variance. Basically, the farther apart the two samples were in the original (31-dimensional) space, the farther apart they were in 2-dimensional space. Differences in CLPPs between samples were tested statistically by comparing principal component scores among different samples using analysis of variance. The plots of PC scores for each sample were used to display differences in the metabolic diversity pattern. Correlation analysis was used to relate original variables (substrates) to the principal components. Interpretation of the principal components was based on significant factor loading of the individual substrates on each of the principal components [17].

All statistical analyses were executed using Minitab 17 statistical software (Minitab Inc., State College, PA).

## 3. Results

### 3.1. Weather History and Field Data

The highest mean air temperature was recorded in the summer (27°C), and the lowest was in the winter (10°C). The mean ambient temperature in the fall was 5°C lower than that in the summer (22°C), while the average ambient temperature in the winter was similar to that in the spring (11°C). The highest mean humidity was recorded in the summer (84%), and the lowest was in the spring (65%). None of the sampling days had any precipitation. Pressure was highest in the winter (771 mmHg) and lowest in the summer (760 mmHg). The visibility was lowest in the fall at 11.27 km, and in the summer and winter, the visibility was at its highest at 16.09 km.

Water temperature at the sites correlated with the ambient temperature, with the highest temperature being observed in the summer and the lowest in the winter sampling. The average water temperature for the spring was 12°C, summer was 25°C, fall was 19°C, and winter was 7°C (Table 1). The water temperature between the river and lake sites was similar at each sampling season. The water depth remained fairly consistent throughout the year, with the shallowest in the site LC2 (0.31 m to 0.76 m) and the deepest in the site SC1 (2.31 m to 3.05 m). In general, the river sites were deeper than the lake sites. The sediment height varied between 172 and 174 m. The water clarity data were measured in the summer, fall, and winter (Table 1). Turbidity readings were higher in the sites SC2 (1.19 m to 1.49 m) and LC3 (1.22 m) than those of the sites SC1 (0.31 m), SC3 (0.31 m), LC1 (0.76 m to 0.91 m), and LC2 (0.61 m). Water clarity remained consistent at each site at different seasons. The pH of the water was relatively consistent across all seasons (Table 1), with the lowest pH being 7.1 (at LC2 in the summer) and the highest being 8.9 (at SC2 in the summer) (Table 1).

### 3.2. Physicochemical Properties of the Sediments

Sediment samples from the Saint Clair River had relatively higher moisture contents than those from Lake Saint Clair, particularly those from the sites SC1 (72 to 102%) and SC3 (48 to 95%) (Table 2). Moisture contents of the sediments from the river sites were highest in the spring, with the site SC1 reaching as high as 102%. Summer and winter moisture contents for the three sites (SC1, SC2, and SC3) were similar (24 to 72% and 19 to 78%, resp.). The moisture contents between the lake sites (19 to 68%) and the river sites (22 to 102%) also varied considerably. Moisture contents of the sediments from all three lake sites (LC1, LC2, and LC3) were stable in the spring (22 to 68%), summer (31 to 56%), and fall (38 to 45%) but dropped significantly during the winter sampling (19 to 20%). It should also be noted that these variabilities are due to differences in the sediments' texture. For instance, sediment samples with high clay contents (SC1, SC3, and LC3) held more water and thus had higher moisture contents than sediment samples with higher sand contents (SC2, LC1, and LC3) (Table 2).

The pH ranges for the sediment extracts (1 : 10 sediment-water ratio) from the river and lake sites were very similar (6.29–7.32 for river sites; 6.52–7.23 for lake sites) and varied little between seasons. The average pH for all sites in the spring, summer, fall, and winter was 6.77, 7.00, 6.99, and 6.81, respectively (Table 2). These pH values were lower than the water pH of the sites at the time of sampling. The seasonal water pH averages for all sites were 8.10 in the spring, 7.90 in the summer, 8.26 in the fall, and 8.01 in the winter (Table 1). Regardless of the lower sediment pH values, the pH of the sediment extracts was similar between seasons.

The electrical conductivity of the extracts (1 : 10 sediment-water ratio) varied significantly between the river and lake sediments (Table 2). The electrical conductivity reading of the extracts from the river sediments ranged between 20 and 258 *µ*S·cm^−1^, while that of the lake sediments fluctuated between 25 and 225 *µ*S·cm^−1^. The highest electrical conductivity reading (258 *µ*S·cm^−1^) was from sediment extracts in the summer at the site SC1, while the lowest (20 *µ*S·cm^−1^) was in the winter at the site SC2. The average conductivity for the spring across all sites was 152 *µ*S·cm^−1^, 119 *µ*S·cm^−1^ for the summer, 131 *µ*S·cm^−1^ for the fall, and 89 *µ*S·cm^−1^ for the winter (Table 2).

The sediments from the river sites tended to have higher organic matter contents than those from the lake sites, particularly from the sites SC1 and SC3, which ranged from 4.94% to 67.5% (Table 2). In general, the organic matter contents were found to be highest in the spring, especially in the sediment samples from the sites SC1 (40.5%), SC3 (67.5%), and LC3 (24.63%). As the seasons went on, the organic matter contents of the sediments declined in all sites from an average reading of 31.59% in spring to 2.68% by winter sampling. This decline in organic matter contents coincided with increases in ash contents, which reached their peaks in the winter (Table 2).

**Figure 2:**
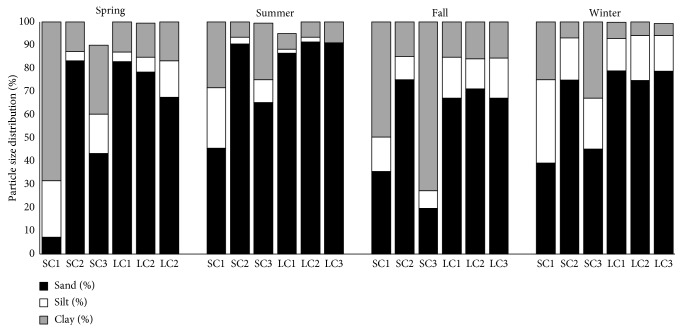
Particle size distribution of the river and lake sediments at different seasons.

The sediments from the three lake sites (LC1, LC2, and LC3) and one from the river site (SC2) consisted mainly of sand (Figure 2). The percentage of sand in these sites varied between 83% and 91% in the spring, with the highest percentage of sand being observed in sediments from the site LC2 (Figure 2). As the seasons went on, the percentage of sand decreased slightly and remained between 75% and 79% at the end of the sampling period (winter season). The two river sites (SC1 and SC3) had relatively more clay (24 to 73%) compared to the lake sites (5 to 17%). The fluctuations in the relative particle size distribution over time were more pronounced in the river sites than the lake sites (Figure 2) due to mixing of the surface sediments. Since lakes are lentic ecosystems (still water habitats), there was little or no mixing of the surface sediments, unlike the river ecosystems (running water habitat) in which the surface sediments are constantly mixed.

### 3.3. Microbial Counts and Dehydrogenase Activities

**Figure 3:**
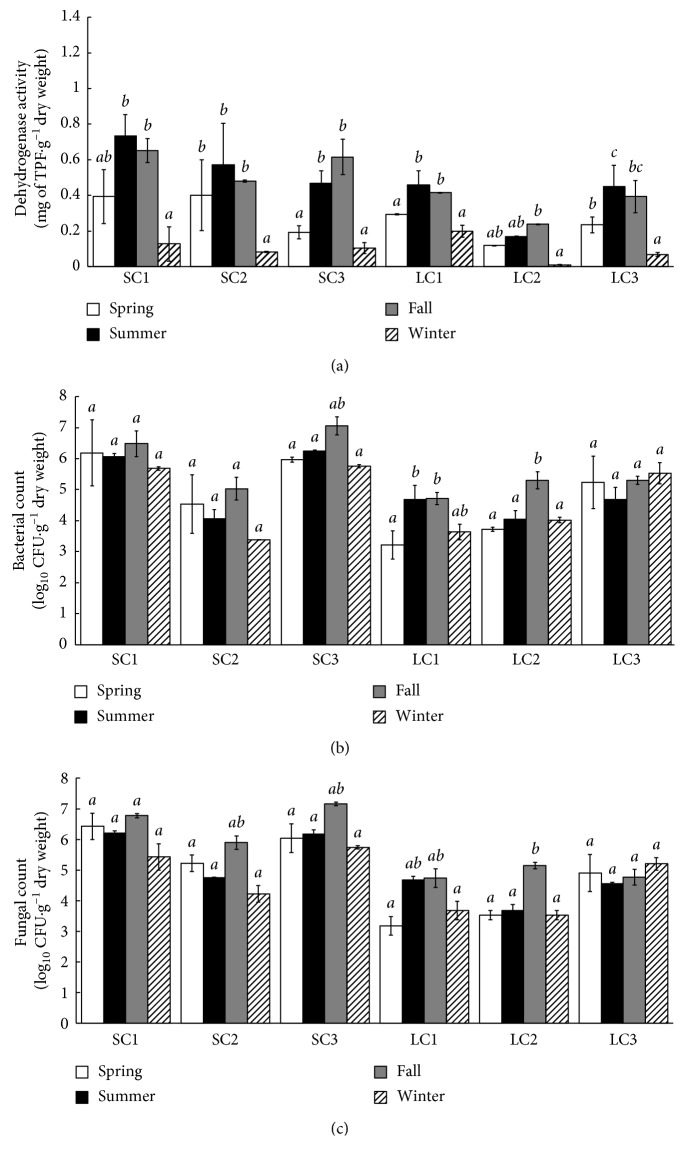
Dehydrogenase activities (a) and bacterial (b) and fungal (c) numbers of the river and lake sediments across different sites and seasons.

Dehydrogenase activity was generally higher in the river sediments than in the lake sediments (Figure 3(a)). The average activity of the lake sites across all seasons was 0.25 mg of TPF·g^−1^, whereas that of the river sites was 0.40 mg of TPF·g^−1^. The sediments had an average dehydrogenase activity value of 1.63 mg of TPF·g^−1^ in the spring, 2.85 mg of TPF·g^−1^ in the summer, 2.79 mg of TPF·g^−1^ in the fall, and 0.59 mg of TPF·g^−1^ in the winter (Figure 3(a)). This result suggests higher decomposition of organic matter of the microbial communities in the summer and fall than in the spring and winter. The dehydrogenase activity test is based on the principle that dehydrogenase enzymes are produced by all living cells and the extent to which this enzyme group oxidizes organic matter can be related to the number of live cells present [43, 44]. This group of enzymes transports electrons and hydrogen through a chain of intermediate electron carriers to a final electron acceptor (oxygen), resulting in the formation of water [42].

Sediments that consisted mainly of sand (LC1, LC2, LC3, and SC2) had lower bacterial heterotrophic numbers than sediments that contained more clay (SC1 and SC3) (Figures 2 and 3(b)). The bacterial numbers in these sandy sites varied between 1.66 × 10^3^ CFU·g^−1^ and 1.07 × 10^5^ CFU·g^−1^ (Figure 3(b)). The heterotrophic bacterial counts of the river sediments were higher (3.39 × 10^4^ CFU·g^−1^ to 1.7 × 10^7^ CFU·g^−1^) than those of the lake sediments (1.66 × 10^3^ CFU·g^−1^ to 3.39 × 10^5^ CFU·g^−1^). The total average CFU·g^−1^ for the lake across all sites was 1.32 × 10^5^ CFU·g^−1^, whereas the average for the river across all seasons was 3.80 × 10^2^ CFU·g^−1^. The average heterotrophic bacterial counts between seasons ranged between 4.68 × 10^4^ CFU·g^−1^ and 4.47 × 10^5^ CFU·g^−1^, with the highest average bacterial count being observed in the fall (Figure 3(b)). The river sediment samples had higher average heterotrophic bacterial counts (3.55 × 10^5^ CFU·g^−1^) than the lake sediment samples (3.24 × 10^4^ CFU·g^−1^) (Figure 3(b)). Sediment samples with higher clay contents (the sites SC1 and SC3) had the highest heterotrophic bacterial numbers (1.25 × 10^6^ CFU·g^−1^ to 1.82 × 10^6^ CFU·g^−1^) (Figure 3(b)).

The fungal counts of the lake sediments (LC1, LC2, and LC3) were generally lower than those of the river sediments (SC1, SC2, and SC3). The fungal numbers of the lake sediments over time showed an average log_10_ CFU·g^−1^ value of 4.47 × 10^2^, while the river sediments had an average of 1.86 × 10^4^ CFU·g^−1^ (Figure 3(c)). These numbers fluctuated for both ecosystems across seasons, with both reaching their peak growth in the summer (lake sediments, 4.90 × 10^4^ CFU·g^−1^; river sediments, 9.77 × 10^5^ CFU·g^−1^). The average fungal counts between seasons were similar and ranged between 1.0 × 10^3^ and 4.17 × 10^3^ log_10_ CFU·g^−1^. Sediments from the sites SC1 and SC3 had higher fungal numbers (4.17 × 10^3^ to 2.04 × 10^4^ CFU·g^−1^) than SC1, LC1, LC2, and LC3 (1.55 × 10^2^ to 2.69 × 10^3^ CFU·g^−1^) (Figure 3(c)).

### 3.4. Community-Level Physiological Profiles (CLPPs)

**Figure 4:**
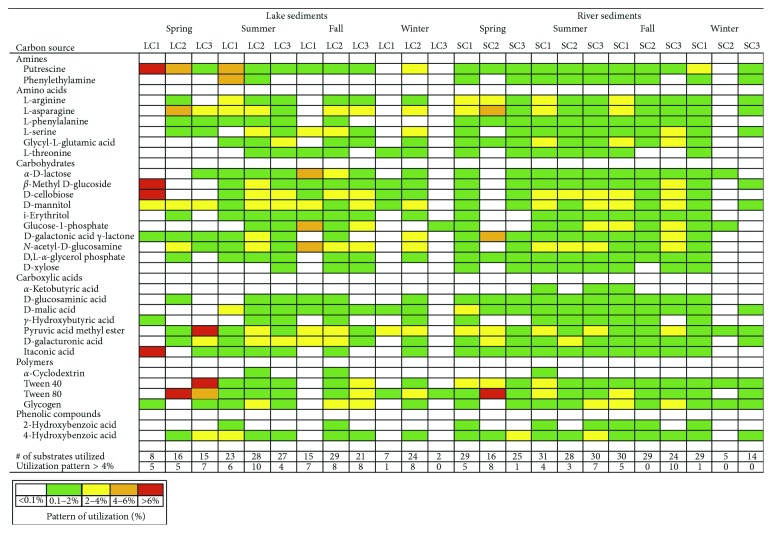
Pattern of utilization (based on mean AWCD) of the 31 carbon substrates for the lake and river sediments across sites and seasons. Shading in the boxes indicates the range of percentage absorbance of the total absorbance of the plate. Values are as follows: white, <0.1%; green, 0.1–2%; yellow, 3-4%; orange, 5-6%; red, >6%. The number of substrates with >4% absorbance for each sampling site is indicated below each column.

In this study, the 31 carbon sources were pooled to the total utilization responses within these six functional classes, including amines, amino acids, carbohydrates, carboxylic acids, polymers, and phenolic compounds (Figure 4). The measured carbon utilization in the sediments differed among substrates in the Biolog EcoPlates and revealed differences and similarities between sediment types (river and lake sediments), seasons (spring, summer, fall, and winter sediments), and sites (SC1, SC2, SC3, LC1, LC2, and LC2 sediments). The number of carbon utilized by the microbial communities varied from 5 to 31 at each site (Figure 4), and the relative abundance based on AWCD for each substrate was also different. Substrates that produced consistently low relative responses in the sediments were probably poorly degraded by the microbial communities in this study. Nonetheless, most substrates in the Biolog EcoPlates seemingly supported at least some bacterial activity. In the present study, we considered 3-4% absorbance of the total absorbance per plate as the threshold for substrate utilization.

### 3.5. Analysis of Substrate Utilization Data from Lake Saint Clair and Saint Clair River Sediments

Inspection of the CLPPs suggests a wide variability in terms of the number of carbon substrates utilized by the river (5 to 31) and lake sediment (2 to 29) microbial communities (Figure 4). Carbon utilization varied considerably between seasons. In the lake sediment samples, more carbon substrates were utilized in the summer (23 to 28) and fall (15 to 29) than in the spring (8 to 15) and winter (2 to 24). In the river sediment samples, carbon utilization in spring, summer and fall was similar (24 to 31) and much higher than the winter sediment samples, particularly in the site SC2 in which only five carbon sources were utilized (Figure 4). *T*-test results showed significant difference in the carbon substrates utilization between the lake and river sediment samples, with respect to only four carbon substrates: phenylethylamine (an amine; *P*=0.045), L-threonine (an amino acid; *P*=0.01), D,L-*α*-glycerol phosphate xylose (a carbohydrate; *P*=0.015), and D-malic acid (a carboxylic acid; *P*=0.042). These four carbon substrates were utilized at a significantly higher level in the river sediments and were either not utilized or poorly degraded by the microbial communities in the lake sediments. Interestingly, *α*-ketobutyric acid (a carboxylic acid) was not utilized by the microbial communities in both ecosystems except in sediments from the sites SC2 and SC3 (Figure 4).

### 3.6. Comparison of Substrate Utilization Data at Different Sites

The breakdown of the percent utilization of the carbon sources into six substrate guilds showed very similar use among the three lake sites (LC1, LC2, and LC3) (Figure 4). A significant difference between the three lake sites was found only on eight of the 31 carbon substrates: L-asparagine (ANOVA, *F* = 3.2, *P*=0.044), D-galacturonic acid *γ*-lactone (ANOVA, *F* = 5.09, *P*=0.012), *N*-acetyl-D-glucosamine (ANOVA, *F* = 3.0, *P*=0.034), *γ*-hydrobutyric acid (ANOVA, *F* = 5.9, *P*=0.066), *α*-cyclodextrin (ANOVA, *F* = 9.2, *P*=0.001), Tween 40 (ANOVA, *F* = 4.2, *P*=0.024), Tween 80 (ANOVA, *F* = 8.5, *P*=0.0001), and glycogen (ANOVA, *F* = 2.5, *P*=0.046). These substrates were shown to be utilized in significantly lower concentration in the site LC1 than in the sites LC2 and LC3. Interestingly, phenylethylamine and 2-hydroxybenzoic acid were not utilized in the site LC3, and no utilization was observed for D,L-*α*-glycerol phosphate, D-xylose, and *α*-cyclodextrin in the site LC1 (Figure 4).

Utilization patterns of the 31 carbon sources in the sediments from the river sites (SC1, SC2, and SC3) were similar to each other except for 11 carbon substrates: phenylalanine (ANOVA, *F* = 9.6, *P*=0.001), L-arginine (ANOVA, *F* = 4.2, *P*=0.23), L-threonine (ANOVA, *F* = 3.7, *P*=0.037), D-galacturonic acid *γ*-lactone (ANOVA, *F* = 5.7, *P*=0.007), D-xylose (ANOVA, *F* = 6.2, *P*=0.005), *α*-ketobutyric acid (ANOVA, *F* = 6.1, *P*=0.006), D-malic acid (ANOVA, *F* = 6.7, *P*=0.004), itaconic acid (ANOVA, *F* = 3.5, *P*=0.040), *α*-cyclodextrin (ANOVA, *F* = 5.6, *P*=0.008), 2-hydroxybenzoic acid (ANOVA, *F* = 7.4, *P*=0.002), and 4-hydroxybenzoic acid (ANOVA, *F* = 10.8, *P*=0 < 0.0001). The analysis of variance (ANOVA) indicated that the utilization of these 11 carbon sources was significantly lower in sediments from the site SC2 than in sediments from the sites SC1 and SC3, and the utilization in the sites SC1 and SC3 was similar. Surprisingly, *α*-ketobutyric acid and 2-hydroxybenzoic acid were not utilized in the site SC2, and no utilization was observed for *α*-cyclodextrin in the sites SC1 and SC3 (Figure 4).

### 3.7. Seasonal Patterns of Substrate Utilization

The pattern of carbon utilization of the heterotrophic communities in sediments varied with season. The number of carbon substrates utilized was less in the spring (8–16 for lake sediments; 16 to 29 for river sediments) than in the summer (23 to 28 for lake sediments; 25 to 31 for river sediments) and fall (15 to 29 for lake sediments; 24 to 30 for river sediments) seasons. The winter season had the least number of carbon utilized (2 to 24 for lake sediments; 5 to 29 for river sediments) (Figure 4). ANOVA results indicated significant seasonal differences for 19 different carbon substrates including 3 amino acids (L-phenylalanine, L-serine, and glycyl-L-glutamic acid), 9 carbohydrates (*α*-D-lactose, *β*-methyl D-glucoside, D-cellobiose, D-mannitol, i-erythritol, glucose-1-phosphate, D-galacturonic acid *γ*-lactone, N-acetyl-D-glucosamine, and D-xylose), 4 carboxylic acids (*α*-ketobutyric acid, *γ*-hydroxybutyric acid, pyruvic acid methyl ester, and D-galacturonic acid), 2 polymers (*α*-cyclodextrin and 2-hydroxybenzoic acid), and 1 phenolic compound (2-hydroxybenzoic acid). The utilization of these 19 carbon sources was significantly higher in the summer and fall sediments than in the spring and winter sediments. Notably, neither *α*-cyclodextrin nor *α*-ketobutyric acid was utilized in the sediments in the spring (Figure 4).

**Figure 5:**
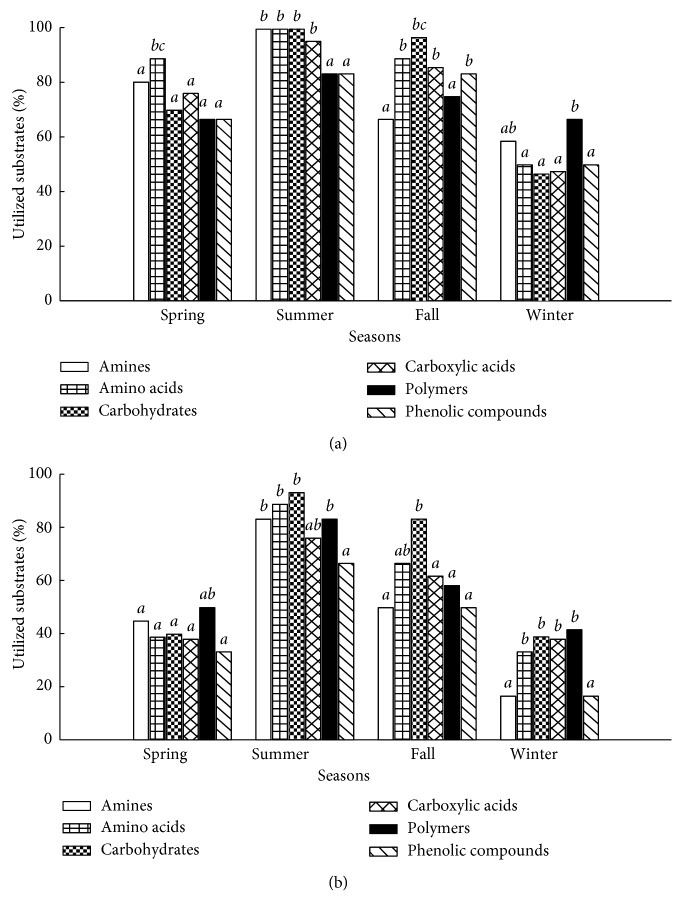
Percent of positive substrates separated into substrate categories for (a) river and (b) lake sediment samples at different seasons.

The substrate utilization patterns in the sediments at different seasons were compared based on substrate categories including various amino acids, amines, polymers, carbohydrates, carboxylic acids, phenolic compounds, and polymers (Figure 5). In general, the lake and river sediment microbial communities in the summer and fall preferred to use carbohydrates. However, the river sediments also used amine and amino acids largely (Figure 5(b)). In the winter, polymers were used most extensively in lake and river sediments. In the spring, polymers were the preferred carbon source in the lake sediments (Figure 5(a)), while it was amino acids for the river sediments (Figure 5(b)).

### 3.8. Multivariate Analysis

**Figure 6:**
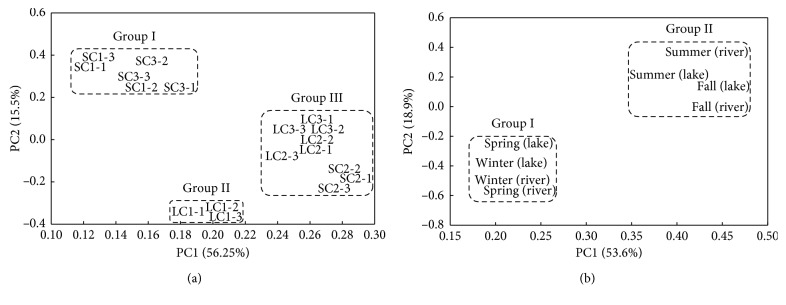
Ordination diagrams of CLPPs from principal component analyses of carbon utilization profiles of lake and river samples from different sites (a) and different seasons (b).

Principal component analysis was performed on binary-transformed data. The first PCA was carried out for the different sites (LC1, LC2, LC3, SC1, SC2, and SC3) from 124 Biolog data sets (Figure 6(a)). The two principal components (PCs) accounted for 72% of the total variance. PC1 and PC2 separated sites into three groups (Groups I, II, and III). Group I consisted of sites (SC1 and SC3) with the higher number of positive substrate utilization. Group II comprised a site (LC1) with the lowest number of positive substrate utilization. Group III consisted of the rest of the sites. PC2 also separated the sites into the same three groups (Figure 6(b)).

PCA was also carried out for the different seasons (spring, summer, fall, and winter) (Figure 6(b)). PC1, which accounted for 53.6% of the variance, and PC2, which accounted for 18.9% of the variance, separated the spring and winter sediment samples (Group I) from the spring and summer samples (Group II) (Figure 6(b)). To relate the utilization of individual carbon sources to the differences in sole carbon utilization patterns, the correlation between the substrates variables and PCs was determined. A high correlation indicates that the variance in the utilization of the carbon source was highest between samples. For PC1, the variability was explained among utilization of amines, amino acids, carbohydrates, carboxylic acids, polymers, and phenolic compounds (Table 3). The variability in PC2 was explained primarily by responses of amines, carbohydrates, carboxylic acids, and phenolic compounds (Table 3).

## 4. Discussion

Lakes and rivers represent dynamic systems with highly variable conditions on both temporal and spatial scales. They collect terrestrial inputs via water and transport them to coastal zones and therefore serve as links between terrestrial landscapes and the oceanic ecosystem [17, 45, 46]. Often, lakes and rivers serve as the main sources of municipal, agricultural, and industrial water for residents living in the basin. However, these freshwater ecosystems are often subjected to pollution because of human activities. It is known that pollution from anthropogenic sources can result in the reduction of changes in the activity, structure, and diversity of the indigenous microbial communities, which is partly linked to the disappearance of more sensitive microbial species [22, 47]. The study presented here attempted to monitor metabolic diversity in river and lake sediments using a phenotypical approach (Biolog EcoPlates), and the potential of this technique is to detect potential shifts in sediment microbial communities by utilizing using organic compounds as they affect the whole ecosystem function.

The microbial communities of the river and lake sediments were capable of using a wide range of compounds after 72 h of incubation. Despite the differences in the physicochemical properties of the river and lake sediments, visual observation of the CLPP fingerprints showed a very little difference between the two sediment samples (Figure 4). Of the 31 carbon substrates, differences were found only on four carbon substrates: phenylethylamine, L-threonine, D,L-*α* glycerol phosphate xylose, and D-malic acid. These four substrates were utilized at a significantly higher level in the river sediments and were either not utilized or poorly degraded by the microbial communities in the lake sediments. Although the *t*-test did not appear to be sensitive enough to define functional differences between the two sediment types (river and lake sediments), when looking for the overall specific carbon sources used, differences in the functional fingerprints can be observed (Figure 4). Overall, the number of metabolized substrates was higher in the river sediments than in the lake sediments (Figure 4). Moreover, the river sediments had a shorter lag time (<24 h) than the lake sediments (>24 h), indicating that the carbon substrates were more rapidly oxidized in the river sediments. The lower number of positive catabolic responses to the carbon substrates in the lake sediments is probably due to the increased accumulation of pollutants in Lake Saint Clair, which led to the substrates being poorly degraded by the lake sediment microbial communities. The sediments in Lake Saint Clair serve as the sink for anthropogenic inputs that enter the lake via the Saint Clair River and the tributary streams. The higher functional diversity implicated in the river sediments was due to its physical, chemical, and biological properties. In general, the river sediments were characterized by a higher utilization of carbon substrates with AWCD values higher than the lake sediments. The river sediments also had higher organic matter, bacterial and fungal density, and dehydrogenase activities than the lake sediments.

Our data showed that the microbial community-level physiological profiles were seasonally different. The numbers of positive substrates were similar in the summer and fall and distinguishable from those in the winter and spring. In the winter, which showed the lowest ambient and water temperatures, the catabolic activities of the microbial communities in sediments across all sites decreased. For example, *N*-acetyl glucosamine was the preferred carbon source in the summer and fall, but the degradation ability was weak in the winter. *N*-acetyl glucosamine is a major component of chitin primarily produced by insects, fungi, and zooplankton. *N*-acetylglucosamine and its decomposition products: glucosamine, acetic acid, and ammonia, are released during the process of chitin decomposition [48]. They are used as a source of carbon and nitrogen by many different organisms in the water column and sediments [48]. The overall CLPPs showed that the metabolic diversity was significantly enhanced from the summer (when ambient and water temperatures were the highest) to fall season, but it dropped rapidly in the winter (when ambient and water temperatures were the lowest). Similar results were found by Urakawa et al. [49] who reported that bacterioplankton assemblages in the summer and fall showed more versatile substrate utilization patterns than winter and spring communities.

The carbon utilization patterns of the sediment communities at each site were also different, suggesting the complexity of the substrate utilization patterns among the different sites. PCA based on carbon substrate utilization indicated that carbohydrates, polymers, amines, and amino acids contributed to the significant variation in increased and/or decreased carbon substrate utilization between the lake and river sediment samples. It is clear that the lake sites utilized carbohydrates and polymers the most, while the river utilized a variety of carbon substrates including amine, amino acids, carbohydrates, and polymers at various extents throughout the seasons. In the spring and summer, the microbial assemblages used nitrogen-rich carbon sources (amino acids) [50] more frequently over carbohydrates and carboxylic acids. These microbial communities utilized amino acids as the nitrogen source by incorporating the ammonium side chain in addition to using carbon. Subsequently, the ammonium is then made into organic molecules such as amino acids and proteins [51]. The high utilization of amino acids in the spring implies that the river sediment microbial communities may be deprived of nitrogen. Nitrogen deprivation in sediments results from decreased sinking of nitrogen-rich organic matter due to lack of reservoir mixing [52, 53]. As summer progressed, as indicated by warm temperatures (Table 1), the substrate preference shifted to amines, amino acids, and carbohydrates (Figure 5(b)). At the start of the fall over turn marked by decreasing temperatures, there were a decreased use of amino acids and amines and a continued little use of carbohydrates. The highest carbohydrate utilization during the fall may suggest that the river sediment communities preferred carbohydrate substrates. Undoubtedly, carbohydrates are the preferred catabolic pathway of many aerobic and facultative heterotrophs, and the catabolic process involves oxidation of a simple or complex carbohydrate and oxygen as a terminal electron acceptor [17, 50, 54–56]. Furthermore, fixed carbon in the form of carbohydrates synthesized by photosynthetic microorganisms and carbohydrate-rich allochthonous organic matter from the water column trickles down and sinks to the sediments during river mixing [57–59]; hence, conditions present during winter mixing may select for river sediments microbial populations that readily utilize carbohydrates. Throughout winter, the river sediment communities had a high preference for polymers.

The lake sediments, on the contrary, preferred more polymers (Tween 40, Tween 80, glycogen, and *α*-cyclodextrin) in the spring, instead of amino acids. Polymers are complex carbon substrates. For instance, Tween 40 and Tween 80 are nonionic surfactants often used as oil-in-water emulsifiers in pharmaceuticals, cosmetics, and cleaning compounds. Glycogen is a highly branched polysaccharide that consists of glucose units linked in a linear chain by *α*-1,4 bonds with *α*-1,6 branches. Glycogen has been shown to accumulate in bacteria upon entry into the stationary phase, or when growth is inhibited due to limitation of required nutrients (e.g., nitrogen or phosphate) in the presence of excess carbon [60]. As these complex organic carbons are degraded into simple ones (e.g., carbohydrates), the carbon utilization shifted to carbohydrates in the summer and fall. In the winter, the lake sediment communities utilized more polymers as more newly discharged complex organic carbon compounds settled into the sediments from the Saint Clair River. The higher use of these two groups (carbohydrates and polymers) of substrates might be related to the available carbon sources in the sediments through carbon deposition. Although the use of a specific carbon substrate from the EcoPlates does not mean that this substrate was available in the sediments, the appearance of specific capabilities suggests that similar strata may exist in the collecting sediment site. For example, the higher use of Tween 40 and 80 might be linked to the presence of pharmaceuticals specifically measured in other studies at this site [61, 62].

The similarity in carbon utilization profiles between the river sites SC1 and SC3 and the lake sites LC2 and LC3 (Figure 6) could be related to possible selection of microbial communities adapted to the specific quality and availability of organic compounds in the sediments [63, 64]. This similarity decreases the spatial heterogeneity in organic matter use capabilities in these sediments. In contrast, the river site SC2 (the middle site) was more difficult to characterize due to the larger heterogeneity between replicate samples. The site had cobbles and gravel, while the rest were covered with sediments.

The dehydrogenase activities and microbial counts correlated with the carbon utilization patterns. Higher dehydrogenase activities and microbial numbers were also noted in river sediments than in lake sediments. The higher carbon utilization in the summer and fall season coincided with higher dehydrogenase activity and microbial counts, although a more consistent trend was more evident for dehydrogenase activity than the bacterial and fungal densities. It needs to be emphasized that the estimation of total aerobic heterotrophic bacteria and fungi in sediments by culture methods has some limitations since many microorganisms from the environment will not grow. The low cultivability and the incubation limitations of cultural methods using environmental samples are well known, and the limitations should be considered when the results are interpreted.

In conclusion, this study has shown that sediments with different biogeochemical properties have microbial communities that exhibit distinct catabolic responses to a range of carbon sources. The sediments in the river site SC1 (upstream) had the highest functional diversity, where the microbial communities were able to use a broader range of substrates (29–31 carbon substrates). The functional fingerprint analyses clearly distinguished the lake sediment microbial communities (more polluted sites) from the river sediment communities by their capacity to use more complex carbon sources such as polymers, which may be linked to differences in the water quality. All data (EcoPlate, microbial counts, and enzyme activity) showed that river sediment communities were more active than lake sediment communities. This study also establishes a close linkage between physicochemical properties (temperature and organic matter content) of the sediments and the associated microbial functional activities. The Biolog EcoPlate assay has been demonstrated to be effective at detecting spatial and temporal changes in metabolic capabilities of the sediment communities and provides information regarding the physiological profiles of the restricted group of culturable microorganisms that may quickly respond to future pollution events.

## Figures and Tables

**Table 1:** Water temperature, water depth, sediment height, water clarity, and water pH.

Sites	Water temperature (°C)	Water depth (m)	Sediment height (m)	Water clarity (m)	Water pH
*Spring*					
Saint Clair River sites					
SC1	11.5	3.05	172.2	ND	8.7
SC2	10.2	0.91	174.3	ND	8.2
SC3	12.9	1.22	170.0	ND	7.9
Lake Saint Clair sites					
LC1	12.4	1.07	174.2	ND	7.9
LC2	15.5	0.76	174.2	ND	7.9
LC3	12.3	0.52	174.2	ND	8.0

*Summer*					
Saint Clair River sites					
SC1	25.0	3.05	172.2	031	7.9
SC2	23.9	1.19	172.2	1.19	8.9
SC3	22.2	1.31	172.2	0.31	8.2
Lake Saint Clair sites					
LC1	22.8	0.76	174.8	0.76	7.3
LC2	25.6	0.61	174.9	0.61	7.1
LC3	28.9	1.31	174.2	1.22	8.0

*Fall*					
Saint Clair River sites					
SC1	22.9	2.35	173.2	0.31	7.7
SC2	21.3	1.49	174.0	1.49	8.7
SC3	20.6	1.41	173.1	0.31	7.7
Lake Saint Clair sites					
LC1	20.6	0.91	174.6	0.91	8.7
LC2	15.6	0.31	175.2	1.19	8.7
LC3	15.3	1.80	173.7	0.31	8.8

*Winter*					
Saint Clair River sites					
SC1	5.00	2.68	172.4	0.31	8.6
SC2	6.67	0.91	174.2	1.19	8.1
SC3	8.89	1.95	173.1	0.31	7.9
Lake Saint Clair sites					
LC1	7.22	0.91	174.2	0.76	8.0
LC2	7.78	0.61	174.5	0.61	7.7
LC3	7.22	1.37	173.7	1.22	7.8

ND = no data available.

**Table 2:** Physicochemical properties of the sediments^a^.

Sites	Moisture content (%)	pH (1 : 10 water extract)	Electrical conductivity (*µ*S·cm^−1^)	Ash content (%)	Organic matter (%)
*Spring*					
Saint Clair River sites					
SC1	102 ± 16.3	6.64 ± 0.02	228 ± 17.1	36.67 ± 40.57	64.33 ± 40.46
SC2	22 ± 2.2	6.29 ± 0.14	33 ± 5.0	86.40 ± 16.84	13.60 ± 16.84
SC3	95 ± 15.0	6.88 ± 0.07	255 ± 20.8	32.54 ± 11.17	67.50 ± 11.17
Lake Saint Clair sites					
LC1	26 ± 0.3	6.53 ± 0.35	90 ± 11.6	96.95 ± 1.568	3.052 ± 1.568
LC2	32 ± 6.2	7.08 ± 0.20	80 ± 0.0	78.65 ± 37.12	21.35 ± 37.12
LC3	68 ± 16.1	7.17 ± 0.29	225 ± 5.8	80.28 ± 24.63	19.72 ± 24.63

*Summer*					
Saint Clair River sites					
SC1	72 ± 81.46	6.67 ± 0.02	258 ± 17.1	67.37 ± 32.58	32.63 ± 32.58
SC2	24 ± 6.50	7.13 ± 0.13	45 ±5.8	99.05 ± 0.36	0.95 ± 0.36
SC3	48 ± 5.80	7.32 ± 0.01	168 ± 5.0	95.21 ± 0.88	4.79 ± 0.88
Lake Saint Clair sites					
LC1	31 ± 0.90	6.68 ± 0.21	73 ± 20.6	98.26 ± 0.99	1.75 ± 0.98
LC2	28 ± 0.54	6.96 ± 0.15	55 ±5.8	98.34 ± 0.10	1.66 ± 0.10
LC3	56 ± 7.41	7.23 ± 0.05	115 ± 5.8	92.46 ± 0.67	7.54 ± 0.67

*Fall*					
Saint Clair River sites					
SC1	81 ± 22.50	6.80 ± 0.05	193 ± 9.6	91.34 ± 1.80	8.66 ± 1.80
SC2	37 ± 7.96	7.10 ± 0.10	63 ± 5.0	98.16 ± 0.09	1.84 ± 0.09
SC3	81 ± 27.34	6.86 ± 0.03	150 ± 0.0	90.89 ± 0.91	9.11 ± 0.91
Lake Saint Clair sites					
LC1	38 ± 10.06	6.78 ± 0.12	160 ± 58.8	97.18 ± 0.80	2.82 ± 0.80
LC2	42 ± 15.89	7.18 ± 0.04	113 ± 9.6	98.27 ± 0.97	1.74 ± 0.97
LC3	45 ± 14.50	7.24 ± 0.10	105 ± 5.8	95.76 ± 1.16	4.24 ± 1.16

*Winter*					
Saint Clair River sites					
SC1	74 ± 23.93	6.70 ± 0.43	185 ± 12.9	95.06 ± 2.05	4.94 ± 2.05
SC2	19 ± 0.84	6.75 ± 0.14	20 ± 0.0	98.81 ± 0.38	1.19 ± 0.38
SC3	78 ± 5.38	6.73 ± 0.06	165 ± 17.3	92.82 ± 1.42	7.18 ± 1.42
Lake Saint Clair sites					
LC1	21 ± 1.85	6.75 ± 0.04	33 ± 5.0	99.36 ± 0.31	0.87 ± 0.29
LC2	19 ± 0.86	7.17 ± 0.27	103 ± 59.67	98.76 ± 0.33	1.24 ± 0.33
LC3	20 ± 1.28	6.77 ± 0.10	25 ± 5.8	99.13 ±0.27	0.64 ± 0.32

^a^Mean and standard deviation of three replicates are shown.

**Table 3:** Correlation coefficients of the first two principal components (PC1and PC2) from the water samples collected at three different sites.

Carbon source^†^	PC1 (*r*)	PC2 (*r*)
*Amines*		
Putrescine	0.701	0.658

*Amino acids*		
L-arginine	0.558	
L-asparagine	0.778	
L-phenylalanine		
L-serine		
Glycyl-L-glutamic acid	0.571	
L-threonine		

*Carbohydrates*		
*α*-D-lactose	0.536	
*β*-Methyl D-glucoside	0.812	
D-cellobiose	0.756	0.878
D-mannitol	0.793	
i-Erythritol	0.5490	0.837
Glucose-1-phosphate	0.736	
D-galactonic acid *γ*-lactone	0.538	
*N*-acetyl-D-glucosamine	0.835	
D,L-*α*-glycerol phosphate		
D-xylose		

*Carboxylic acids*		
*α*-Ketobutyric acid		
D-glucosaminic acid		
D-malic acid		−0.775
*γ*-Hydroxybutyric acid		
Pyruvic acid methyl ester		
D-galacturonic acid		
Itaconic acid		

*Polymers*		
*α*-Cyclodextrin	0.701	
Tween 40	0.618	
Tween 80	0.791	

Glycogen		
*Phenolic compounds*		
2-Hydroxybenzoic acid		
4-Hydroxybenzoic acid		−0.833

All substrates with an *r* value > 0.5 are shown (*P* < 0.001); *r* = Pearson's correlation coefficient.

## Data Availability

All other data arising from this study are contained within the manuscript.
